# Laboratory characteristics of IgG4-related disease: A retrospective study from a single tertiary medical center

**DOI:** 10.1097/MD.0000000000030387

**Published:** 2022-09-09

**Authors:** Hanwool Cho, Jeong Joong Lee, Myungshin Kim, Eun-Jee Oh, Yonggoo Kim, Hyojin Chae

**Affiliations:** a Department of Laboratory Medicine, St. Vincent’s Hospital, College of Medicine, The Catholic University of Korea, Seoul, Republic of Korea; b Department of Laboratory Medicine, Seoul St. Mary’s Hospital, College of Medicine, The Catholic University of Korea, Seoul, Republic of Korea.

**Keywords:** heavy/light chain, IgG4, IgG4-related disease, serum electrophoresis, serum free light chain

## Abstract

Immunoglobulin G4-related disease (IgG4-RD) is an immune-mediated fibroinflammatory condition with unique histopathological features that can affect most organs, making diagnosis challenging. This study characterized detailed laboratory characteristics of IgG4-RD.

Baseline clinical and laboratory features of 33 patients with IgG4-RD were reviewed, including serum IgG4 concentrations, serum free light chains (sFLCs), IgGĸ- and IgGλ-heavy/light chains (HLCs), capillary serum protein electrophoresis (SPE), and immunofixation electrophoresis (IFE) of IgG4 subclass.

The cohort of 33 patients showed male predominance (94%), with 8 (24%) exhibiting multiple organ involvement. Most patients (88%) had an elevated IgG4 concentration, and 67% had elevated erythrocyte sedimentation rate and IgE levels. Median IgG4 concentration at baseline was significantly higher in patients with >2 organs involved than those with ≤2. Furthermore, erythrocyte sedimentation rate was significantly correlated with serum IgG4 concentrations at baseline. SPE results demonstrated polyclonal gammopathy in most patients. Half of the patients had an increased κ/λ sFLC ratio, 42% had an increased IgGκ/IgGλ HLC ratio. Most patients exhibited hypergammaglobulinemia in the anodal end of the ɤ region on SPE.

This study describes detailed laboratory features of IgG4-RD. *Although none of these tests are considered diagnostically sufficient by itself, the provided laboratory characteristics can increase awareness of this disorder and help distinguish it from other IgG4-RD mimics.*

## 1. Introduction

Immunoglobulin G4-related disease (IgG4-RD) was first reported in 2001 in patients with sclerosing pancreatitis and was associated with a high serum concentration of IgG4.^[1]^ Since its first description, extrapancreatic involvement has been shown in virtually every organ system, including the biliary tree, salivary glands, periorbital tissues, kidney, lungs, lymph nodes, meninges, aorta, breast, prostate, thyroid, pericardium, and skin, and IgG4-RD has been recognized as a systemic disease.^[2,3]^ However, the precise pathogenesis of IgG4-RD as well as the role of IgG4 in IgG4-RD remains unclear. The various organs involved in IgG4-RD show unique histopathological features of dense lymphoplasmacytic infiltrate, a storiform pattern of fibrosis, obliterative phlebitis, and increased numbers of IgG4 + plasma cells, which has led to the recognition of the disease as a distinctive clinicopathological entity.^[4]^

However, due to the diversity of organs that can be involved in IgG4-RD, clinical symptoms are highly variable, and concurrent or metachronous multiple organ involvement, seen in 50% of patients, augments the variability of clinical manifestations. Although the histopathological features of IgG4-RD have been defined and incorporated into a guideline for the diagnosis of IgG4-RD,^[5]^ diagnostic sensitivity is not sufficient for patients with lesions that are technically challenging for biopsies, such as those of the pancreas, retroperitoneum, and ocular cavity.^[6]^ Moreover, due to the presence of many IgG4-RD mimickers, such as autoimmune diseases, atopic diseases, and cancers, an increased serum concentration of IgG4 is not specific for IgG4-RD. Therefore, increased awareness as well as broader education of the laboratory features associated with this disease seems essential for the diagnosis of this rare disease with a high degree of mimicry.^[7,8]^ Hence, the aim of this study was to describe and characterize the laboratory characteristics of IgG4-RD at initial diagnosis using cases retrospectively identified from a single tertiary hospital.

## 2. Materials and Methods

### 2.1. Study subjects

We conducted a retrospective analysis of consecutive serum samples submitted for IgG4 subclass assay between May and October 2021 at Seoul St. Mary’s Hospital, a 1500-bed tertiary academic medical center in Seoul, Korea. Of the submitted samples during this period, only those patients with an increased IgG4 concentration (>135 mg/dL) were retained for a review of medical records. Data pertaining to the patient’s demographics and clinical, radiologic, laboratory, and pathologic findings were retrieved from the electronic medical records. The diagnosis of IgG4-RD was based on clinicopathologic correlation and was made according to the Comprehensive Diagnostic Criteria for IgG4-RD.^[6]^ The patient outcomes were assessed and primary outcome was defined as the efficacy of the first-line therapy, and secondary outcome measured the rate of relapse.^[9]^ Relapse of IgG4-RD was defined as symptomatic, radiological, or functional exacerbation of organ involved at initial diagnosis or de novo organ involvement. This study was reviewed and approved by the Institutional Review Board of Seoul St. Mary’s Hospital and was granted a waiver of informed consent (KC21RISI1001).

### 2.2. Laboratory assessments

Serum IgG4 subclass, serum free light chains (FLCs), and the IgGĸ-IgGλ heavy/light chain isotype were measured on an Optilite turbidimetric analyzer (The Binding Site, Birmingham, United Kingdom) with the dedicated reagents. The Optilite analyzer performed automatic dilutions for samples with initial results exceeding the upper limit of the assay measuring range and when reaction kinetics monitoring flagged for potential antigen excess. Serum protein electrophoresis (SPE) and immunotyping were performed with capillary electrophoresis on a Capillarys 3 system (Sebia, Lisses, France). Immunofixation electrophoresis of IgG4 subclass was performed using HYDRASYS 2 SCAN (Sebia) with sheep antihuman monospecific antisera for IgG4 (The Binding Site) as described previously.^[10,11]^ Briefly, 10 μL of prediluted sera (1:3 dilution with designated diluent) was electrophoretically separated in agarose gels (20 W and 20°C for 9 minutes). After electrophoresis, the proteins were precipitated with 25 μL of sheep antihuman monospecific IgG4 antisera and incubated for 5 minutes. After staining (4 minutes with violet blue), destaining (3, 2, and 6 minutes, respectively), and drying (8 minutes at 75°C), the stained gels were visually inspected.

### 2.3. Statistical analysis

Binary variables were expressed as frequencies and continuous variables as means and standard deviations or medians and interquartile ranges (IQRs) as appropriate. Normality was assessed using the D’Agostino–Pearson normality test. A Mann-Whitney test was performed to compare continuous variables. Nonparametric Spearman rank correlation was used to test the correlation of continuous variables. All statistical analyses were performed using MedCalc 20.011 (MedCalc Software, Mariakerke, Belgium), and a 2-tailed *P* value ≤ 0.05 was considered statistically significant.

## 3. Results

### 3.1. Clinical Characteristics

Among the 50 patients whose serum samples were requested for IgG4 subclass measurement and had an elevated IgG4 concentration (>135 mg/dL), 33 patients fulfilled the inclusion criteria. Of the 33 patients, 10 patients were requested at initial presentation. The demographic and clinical characteristics of the 33 patients at baseline are summarized in Table 1.

**Table 1:** Demographic and clinical characteristics of 33 patients with IgG4-related disease at baseline.

**Feature**	**N (%), mean ± SD, median (IQR**)
Male, n (%)	31 (93.9)
Age at initial diagnosis, mean ± SD, y	62 ± 11
Organ manifestation	
Multiorgan involvement, n (%)	8 (24.2)
Single organ involvement, n (%)	25 (75.7)
ESR, mean ± SD, mm/h	35 ± 28
Elevated ESR (>15 mm/h), n (%)	22 (66.7)
CRP, median (IQR), mg/dL	0.14 (0.05–0.25)
Elevated CRP (≥0.5 mg/dL), n (%)	3 (9.1)
Total protein, median (IQR), g/dL	8.0 (7.1–8.5)
IgG, median (IQR), mg/dL	2164 (1468–3271)
IgA, median (IQR), mg/dL	245 (169–292)
IgM, median (IQR), mg/dL	72 (58–102)
IgE, median (IQR), mg/dL	915 (147–1565)
IgG4, median (IQR), mg/dL	511 (207–1414)
Increased IgG4 (>135 mg/dL), n (%)	29 (87.9)
C3, mean ± SD, mg/dL	83 ± 27
C4, mean ± SD, mg/dL	19 ± 8

CRP = C-reactive protein, ESR = erythrocyte sedimentation rate, IgG4 = immunoglobulin G4, IQR = interquartile range, SD = standard deviation.

The cohort showed a very high male to female ratio of 31:2. The mean ± standard deviation age at initial diagnosis was 62 ± 11 years (range 31–86 years). In total, 13 different organs were involved according to the classification of organ manifestations in IgG4-RD as described by Umehara et al.^[12]^ The most common organ manifestations included pancreatitis (18%), retroperitoneal fibrosis (16%), sialadenitis (14%), and ophthalmic disease (11%). Of the 33 patients, 8 (24%) had multiple organ involvement, with 1 patient each, having 3 and 4 organs involved, respectively.

### 3.2. Laboratory findings

The median serum IgG4 concentration at baseline was 511 mg/dL (IQR 207–1414 mg/dL). Most of the patients (31/33) had an elevated IgG4 concentration (>135 mg/dL). Among the patients whose erythrocyte sedimentation rate (ESR) or C-reactive protein (CRP) level was measured at baseline (n = 27 and n = 29, respectively), 67% (22/27) had an elevated ESR and 10% (3/29) had an elevated CRP. The mean C3 and C4 concentration at baseline was 83 and 19 mg/dL, respectively, and hypocomplementemia (C3 < 90 mg/dL and C4 < 10 mg/dL) was observed in 21% (4/19) of the patients. The median serum IgE concentration at baseline was 915 IU/mL (IQR 147–1565 IU/mL) and most (67%) of the patients had elevated IgE levels compared to the reference range (<158 IU/mL).

### 3.3. Organ involvement and total IgG and IgG4 levels

Although there was no significant difference in the serum IgG4 concentrations at baseline between patients with single organ involvement and those with multiple organ involvement, the median IgG4 concentration at baseline of patients with > 2 organs involved was significantly higher than that of patients with ≤ 2 organs involved (4905 mg/dL vs 489 mg/dL, *P* = .030). There was no significant difference in the serum IgG4 concentrations at baseline between patients with and without specific organ manifestations (i.e., pancreatitis, retroperitoneal fibrosis, and sialadenitis). Furthermore, there were no significant differences in the total IgG concentrations at baseline between patients with single organ involvement and those with multiple organ involvement, as well as between patients with ≤2 organs involved and those with >2 organs involved.

### 3.4. Correlation among laboratory markers at baseline

The baseline serum IgG4 concentrations were significantly correlated with ESR (ρ = 0.434, *P* = .030), but there was no significant correlation with CRP, IgE, C3, C4, ĸ FLC, λ FLC, ĸ/λ FLC ratio, IgGĸ- and IgGλ-heavy/light chains, and IgGĸ/IgGλ ratio. Total IgG concentration was significantly correlated with C4 (ρ = -0.676, *P* = .003), ESR (ρ = 0.730, *P* < .001), and CRP (ρ = 0.555, *P* = .003). However, there were no significant correlations between total IgG and the C3 and IgE concentrations.

### 3.5. Serum protein electrophoresis and serum free light chain and heavy light chain results

The results from SPE, serum FLC assay, and IgGĸ-IgGλ heavy/light chain immunoglobulin analysis were available in 10 patients at baseline (Table 2). In most patients, the SPE demonstrated polyclonal gammopathy with associated hypergammaglobulinemia (>2.0 g/dL). In all patients with polyclonal gammopathy, both the κ and λ FLC concentrations were increased above the reference interval (RI, κ FLC 3.30–19.40 mg/L, λ FLC 5.71–26.30 mg/L), with a median of 231.98 and 214.93 mg/L, respectively. Of the 10 patients, 5 patients had an increased κ/λ FLC ratio (RI, 0.26–1.65) with a median of 1.94 (range 1.74–3.48) and 1 (case 9) had a skewed κ/λ FLC ratio toward λ FLC, with a decreased κ/λ FLC ratio of 0.23 and increased κ and λ FLC concentrations of 171.67 and 745.39 mg/L, respectively. There were no significant differences in the ĸ FLC, λ FLC, and ĸ/λ FLC ratios at baseline between patients with single organ involvement and those with multiple organ involvement.

**Table 2:** Free light chain and heavy/light chain immunoglobulin analysis measured at baseline in patients with IgG4-RD.

Case no.	Serum protein electrophoresis	Gamma fraction (g/dL)	κ FLC (mg/L) (RI: 3.30–19.40 mg/L)	λ FLC (mg/L) (RI: 5.71–26.30 mg/L)	κ/λ FLC ratio (RI: 0.26–1.65)	IgGκ (g/L) (RI: 4.03–9.78 g/L)	IgGλ (g/L) (RI: 1.97–5.71 g/L)	IgGκ/IgGλ ratio (RI: 0.98–2.75)	IgG (mg/dL) (RI: 870–1700 mg/dL)	IgG4 (mg/dL) (RI: 3.9–86.4 mg/dL)
1	Polyclonal gammopathy	2.5	106.36	80.55	1.32	ND	ND	ND	2115	242.36
2	Polyclonal gammopathy	2.07	72.54	39.37	1.84	15.72	4.28	3.67	1667	391.1
3	Polyclonal gammopathy	4.32	453.66	233.65	1.94	ND	ND	ND	4855	515.91
4	Polyclonal gammopathy	5.52	386.54	102.34	3.48	ND	ND	ND	5640	7880
5	Polyclonal gammopathy	7.46	319.15	214.93	1.48	58.06	21.46	2.71	6214	1365.71
6	Normal	1.44	20.4	24.11	0.85	10.37	4.93	2.10	1255	161.92
7	Polyclonal gammopathy	3.47	629.74	215.94	2.92	51.59	13.57	3.80	5266	1222.05
8	Polyclonal gammopathy	3.17	153.68	88.38	1.74	37.29	6.13	6.08	3387	1929.4
9	Polyclonal gammopathy	4.75	171.67	745.39	0.23	12.83	22.79	0.56	3936	2644.27
10	Restricted band in gamma region	2.38	231.98	402.66	0.58	42.48	23.47	1.81	6906	511.07

FLC = free light chain, RI = reference interval.

**Figure 1.**
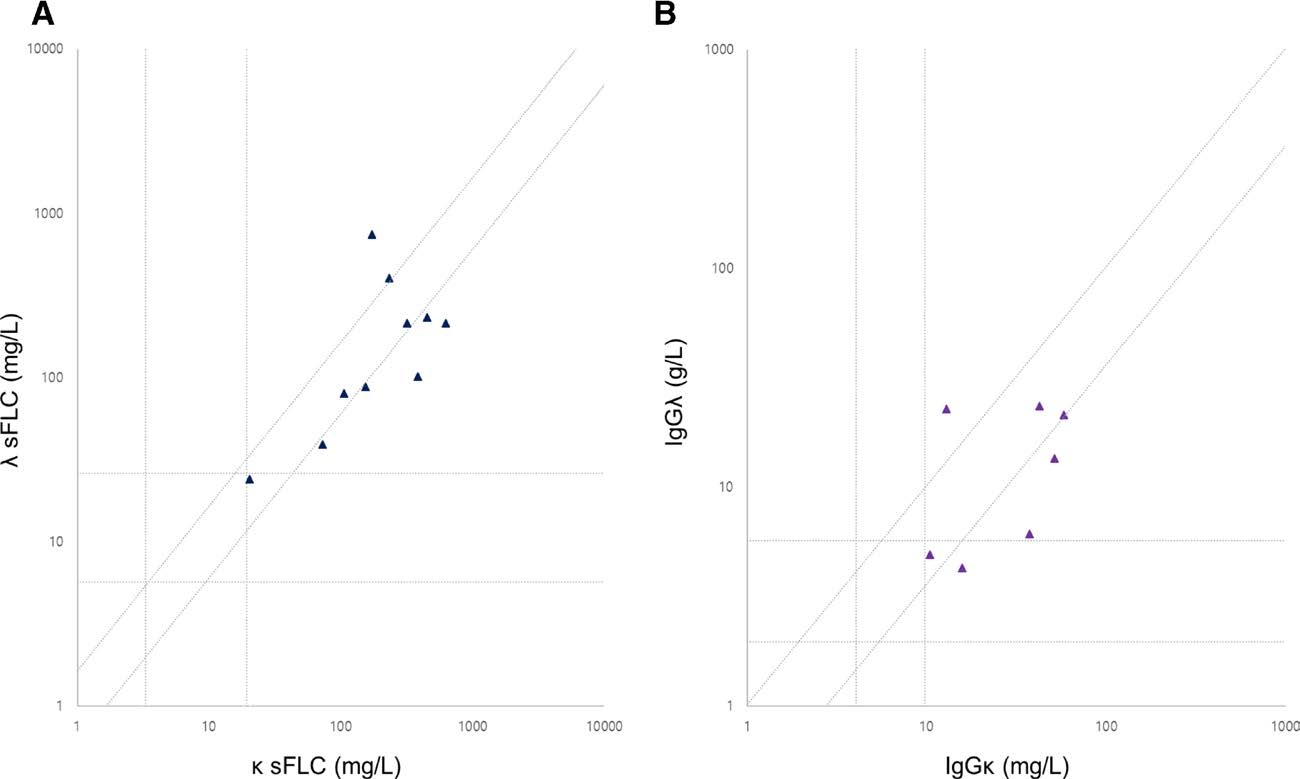
sFLC (A) and IgG ĸ-IgGλ HLC (B) dot plot showing results for patients with IgG4-related disease at baseline. Both axes are represented in logarithmic scale and the broken lines indicate the reference ranges for κ/λ FLCs, IgGĸ-IgGλ HLCs, and their respective ratios. HLC = heavy/light chain, IgG = immunoglobulin G, sFLC = serum free light chain.

Of the 7 patients whose IgGĸ-IgGλ heavy/light chain immunoglobulin levels were measured at baseline, the IgGĸ heavy/light chain concentrations increased above the RI (4.03–9.78 g/L) in all patients, with a median of 37.29 g/L, while the IgGλ heavy/light chain concentrations increased above the RI (1.97–5.71 g/L) in 5 (71%) patients. In addition, 3 (43%) patients had an increased IgGĸ-IgGλ heavy/light chain ratio (RI, 0.98–2.75) with a median of 3.80 (range 3.67–6.08) (Fig. 1), and 1 patient had a decreased IgGĸ-IgGλ heavy/light chain ratio of 0.56.

**Figure 2.**
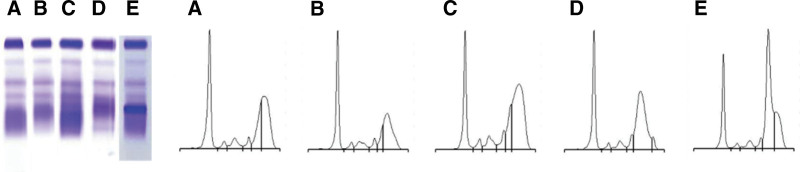
SPE results obtained with agarose gels (on the left) and capillary electrophoresis (on the right) of patient A (A), patient B (B), patient C (C), patient D (D), and patient E (E). In SPE, polyclonal gammopathy with a broad, homogeneous band localized at the anodal end of the gamma region is characteristic of IgG4-RD. A to E represent the SPE patterns of cases 5, 8, 7, 9, and 10 in Table 2. Note that in patient E, in whom monoclonality was excluded during workup, the SPE results strongly mimic a monoclonal band. SPE = serum protein electrophoresis.

**Figure 3.**
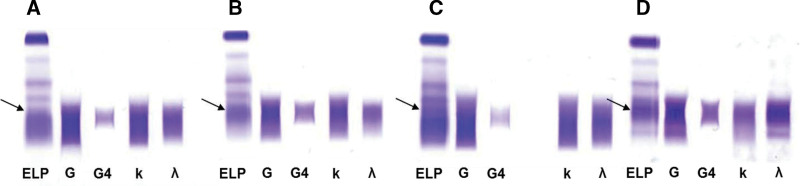
IFE results with anti-IgG, anti-IgG4, anti-κ, and anti-λ sera in patient A (A), patient B (B), patient C (C), and patient D (D) with IgG4-related disease at baseline. IFE with anti-IgG4 sera confirmed the localized migration pattern of IgG4 subclass in the anodal end of gamma region. Panels (A–D) represent the SPE patterns of cases 5, 8, 7, and 9 in Table 2. anti-IgG = anti-immunoglobulin G, ELP = electrophoresis, IFE = immunofixation electrophoresis, SPE = serum protein electrophoresis.

The results of the SPE performed with agarose gel electrophoresis at baseline are shown in Figure 2. In general, an increased staining in the ɤ region, consistent with hypergammaglobulinemia, was seen with relative accentuation in the anodal end of the ɤ region. Interestingly, some cases did show more restricted monoclonal-band like staining than others (Fig. 2D,E, cases 9 and 10). The IFE using sheep antihuman monospecific IgG4 antisera demonstrated the localized electrophoretic migration of IgG4 (Fig. 3).

### 3.6. First-line therapies and treatment outcomes

Among the 32 patients, except for 1 who was lost to follow-up, 29 (91%) received first-line therapy, and 3 (9%) did not receive treatment (Table 3). Most patients (81%, 26/32) were treated with glucocorticoid-based regimen, 1 (3%) was treated with surgery, and 2 (6%) were treated with other therapy (rituximab and antitubercular agents, respectively). Among 27 patients of which records are available to determine the effectiveness of first-line therapy, treatment was effective in 26 patients (96%), and only 1 (4%) patient showed no response to first-line therapy. Of the patients who showed therapeutic response, 25 patients were treated with glucocorticoid-based regimen as first-line therapy, and 1 patient was treated with rituximab as first-line. One patient who showed no response was treated with glucocorticoid-based regimen.

**Table 3:** First-line therapeutic regimens and outcomes.

**First-line therapy**	**N (%**)
Glucocorticoid-based regimens	26 (81.3)
Glucocorticoid alone	24 (75.0)
Glucocorticoid with other therapies	2 (6.3)
Surgery	1 (3.1)
Other therapies*	2 (6.3)
No therapy	3 (9.4)
**Efficacy of first-line therapy**	
Glucocorticoid-based regimens	25/26(96.2)
Glucocorticoid alone	23/24 (95.8)
Glucocorticoid with other therapies	2/2 (100)
Other therapies†	1/1 (100)
**Secondary outcomes**	
Relapses	10 (31.3)
No relapses	22 (68.8)

* Other therapies included rituximab (1) and antitubercular agents (1).

† Other therapies included rituximab.

Ten of the 32 patients (31%) relapsed after treatment. The occurrence of relapse was not associated with the serum IgG4, serum IgG, κ sFLC, λ sFLC, κ/λ sFLC ratio, IgGκ heavy/light chain, IgGλ heavy/light chain, and IgGκ/IgGλ heavy/light chain ratio at baseline (*P* = .404, .917, .643, .758, .440, .770, .770, and .558, respectively).

## 4. Discussion

IgG4-RD has emerged as a relatively novel immune-mediated disorder that can manifest as multiple fibroinflammatory conditions in diverse organ systems, which were previously regarded as separate entities.^[3]^ Although the characteristic histopathological features as well as the elevated number of IgG4 + plasma cells within tissue serve as the gold standard for diagnosis and unify the disparate manifestations, the clinical symptoms vary remarkably depending on the affected organ, resulting in a delayed diagnosis of more than 1 year from presentation, in a significant proportion of patients.^[13]^ An elevated serum IgG4 concentration, which was once considered an essential diagnostic factor of IgG4-RD, has been found to lack both sensitivity and specificity as a disease biomarker.^[1,14,15]^ In a large study involving a 125-patient cohort, composed of biopsy-proven, clinically active IgG4-RD patients, only 51% of patients had elevated IgG4 concentrations.^[16]^ In addition, the reported specificity of elevated IgG4 concentration has been poor at 60% due to the multiple non-IgG4-RD conditions, including chronic sinusitis, recurrent pneumonia, systemic autoimmune disorders, and pancreaticobiliary diseases.^[17]^ Hence, the diagnosis of IgG4-RD is a significant challenge, and an increased awareness of the disorder as well as a greater understanding of the associated laboratory features are important for reducing the delay in diagnosis and decreasing unnecessary surgical interventions, the incidence of which has been reported as high as 40%.^[16]^

In this study, we reported the clinical and laboratory features of 33 Korean IgG4-RD patients at baseline from a single institution. Our cohort was characterized by male preponderance. The reason for this is uncertain, and while IgG4-related pancreatitis is known to show male dominance with a reported M:F ratio of 4:1 to 3:1, other frequently affected organs in our cohort, such as IgG4-related sialadenitis and dacryoadenitis, are known to occur more frequently in females.^[12]^ The male predominance in our study may in part be explained by the inclusion criteria of elevated serum IgG4 concentrations used in this study and may support the concept that fundamental differences exist in the expression of IgG4-RD and higher serum IgG4 concentrations are observed in male patients compared to female patients with IgG4-RD.^[17]^

There was a significant difference in the IgG4 concentration between patients with ≤2 organs involved and those with >2 organs involved. This is in line with previous reports that the IgG4 concentration is higher in multiorgan involvement than that in single organ involvement.^[18,19]^ This suggests that careful investigation of multiorgan involvement is required in patients with high IgG4 concentrations.

Among the inflammatory markers, the ESR level showed significant correlation with the IgG4 concentration, and the ESR, CRP, and C4 levels showed significant correlations with the total IgG concentration. This points to the fact that other IgG subclasses also increase along with the increase in IgG4 in IgG4-RD patients, which is related to inflammatory conditions.^[16]^

It is not unusual during the diagnostic workup of IgG4-RD, especially in cases presenting with lymphadenopathy, for SPE and associated assays to be performed. In most patients in this study, SPE at baseline showed polyclonal gammopathy. What differentiates IgG4-RD from a typical polyclonal gammopathy is the presence of a homogeneous peak that is located at the anodal end of the ɤ region. This is due to the specific migration characteristic of the IgG4 subclass of immunoglobulins, which is also visualized in the IFE results performed in our study with IgG4 antisera.^[11]^

Another distinguishing laboratory characteristic of IgG4-RD is a skewed sFLC and IgGĸ-IgGλ heavy/light chain ratios in a significant proportion of patients at baseline. sFLC overproduction is an indisputable finding in polyclonal gammopathy; however, the κ/λ FLC ratio remains normal under these conditions.^[20]^ However, 50% of patients with IgG4-RD had an increased κ/λ FLC ratio at baseline in this study, and other studies have also observed that 28.9% to 43% of patients have an abnormally increased κ/λ FLC ratio.^[20,21]^ A skewed κ/λ FLC ratio accompanied by a relatively restricted migration on SPE has been reported as a pattern of κ-restricted pseudoclonality and may mimic a monoclonal gammopathy.^[11,22]^ A significantly abnormal κ/λ FLC ratio reflects the excess secretion of 1 light chain type over the other and is considered a surrogate maker of clonality in plasmaproliferative or lymphoproliferative disorders.^[23]^ However, the κ-restricted pattern characteristic of the pseudoclonality of IgG4-RD should not be interpreted as a marker of clonal proliferation but is “pseudoclonal” and results from the difference in the ratio of κ- and λ-expressing IgGs among the IgG subclasses, and the IgG4 subclass has a κ/λ ratio of 8:1.^[22]^ Hence, while sFLC assessment may not be helpful per se for distinguishing polyclonal versus monoclonal gammopathy, it is noteworthy that the median κ/λ FLC ratio in IgG4-RD patients with a skewed κ/λ FLC ratio in this study was 1.96 and ranged from 1.74 to 3.48, which is near the upper limit of a normal κ/λ FLC ratio (1.65). Moreover, compared with the sFLC assay, IgGĸ-IgGλ heavy/light chain assessment may be more useful in distinguishing IgG4-RD from other mimickers. To the best of our knowledge, this is the only study that has assessed IgGĸ-IgGλ heavy/light chain values in patients with IgG4-RD. Our findings showed that the IgGĸ concentrations were increased in all the patients with IgG4-RD, and the IgGĸ-IgGλ heavy/light chain ratio was abnormal in 43% of the patients. Interestingly, the skewed IgGĸ-IgGλ heavy/light chain ratio tended to be more deviated from the normal range (median of 3.80) compared with the κ/λ FLC ratio.

In the present cohort, most patients (91%) were treated and the response to first-line therapy was observed in most of the treated patients (96%). Relapse and recurrent disease were observed in 31% patients (9 treated patients and 1 untreated patient). Previous meta-analysis study reported a similar rate of efficacy of first-line therapies (96%) and relapse (33%).^[9,24]^ In the present study, the occurrence of relapse was not related to laboratory findings at baseline including IgG4, IgG, FLC, FLC ratio, HLC, and HLC ratio. The role of IgG4 as a marker of disease relapse has shown inconsistent results. In line with our result, Mizushima et al have also reported that there was no correlation between baseline serum IgG4 concentration and outcomes in treated patients.^[24]^ However, Mizushima et al reported that baseline serum IgG4 concentration can be predictive of unfavorable outcomes in untreated patients.^[24]^ Also, Culver et al, in a prospective cohort, have reported that baseline serum IgG4 concentration was higher in patients who relapsed after therapy than patients who did not relapse (median 505 mg/dL vs 195 mg/dL).^[19]^ This inconsistency among reports may be due to the different size of the patient population, the design of the study, and different treatment outcomes, and the role of the initial IgG4 level as a marker of disease relapse should be resolved in future studies.

Our study has limitations. First, the retrospective design of this study required that enrolled patients had at least 1 recorded instance of elevated serum IgG4 concentration for entry. Since the number of patients included in this cohort is quite small and only patients with increased IgG4 concentration were included, the cohort of this study is limited in representing the IgG4-RD population, and this may have inevitably introduced bias to the study. In addition, the entirety of laboratory findings analyzed in this study could not be obtained in all patients due to the retrospective design. Second, the clinical and laboratory features were assessed only at baseline, and neither the correlation of disease activity nor a systematic longitudinal follow-up of patients were performed in our study. However, an elevated serum IgG4 concentration can serve as the first recognizable finding of a patient with IgG4-RD from a laboratory perspective, and our study has provided detailed laboratory features including SPE, IFE, sFLC, and IgGĸ-IgGλ heavy/light chain assays.

In conclusion, we have described the laboratory features of patients with IgG4-RD from a single tertiary hospital. None of these tests alone are considered sufficient for the purpose of diagnosis and prediction of outcome. However, the laboratory features associated with IgG4-RD provided in this study can increase the awareness of this disorder in the laboratory community and help to distinguish this disorder from other IgG4-RD mimickers, including monoclonal gammopathy.

## Acknowledgements

We would like to acknowledge Suhyeon Park (SSMedipia Co.) and Jong Deuk Park for their contribution in providing technical support for immunofixation electrophoresis in this study.

## Author contributions

Conceptualization: Hyojin Chae

Data curation: Hanwool Cho, Hyojin Chae

Formal analysis: Jeong Joong Lee, Hanwool Cho, Hyojin Chae

Investigation: Hanwool Cho, Hyojin Chae

Methodology: Hyojin Chae

Project administration: Hyojin Chae

Resources: Jeong Joong Lee, Hanwool Cho, Hyojin Chae, Myungshin Kim, Eun-jee Oh, Yonggoo Kim

Supervision: Hyojin Chae

Validation: Hyojin Chae

Visualization: Hanwool Cho, Hyojin Chae

Writing—original draft: Hanwool Cho, Hyojin Chae

Writing—review and editing: Hyojin Chae, Myungshin Kim, Eun-jee Oh, Yonggoo Kim
